# Titanium Acetabular Component Deformation under Cyclic Loading

**DOI:** 10.3390/ma13010052

**Published:** 2019-12-20

**Authors:** Nicholas A. Beckmann, Rudi G. Bitsch, Theresa Bormann, Steffen Braun, Sebastian Jaeger

**Affiliations:** 1 Clinic for Orthopedics and Trauma Surgery, Heidelberg University Hospital, Heidelberg University, 69118 Heidelberg, Germany; 2 Department of Orthopaedic Surgery and Traumatology, Inselspital, Bern University Hospital, 3010 Bern, Switzerland; 3 National Joint Center, ATOS Clinics, 69115 Heidelberg, Germany; rudi.bitsch@atos.de; 4 Laboratory of Biomechanics and Implant Research, Clinic for Orthopedics and Trauma Surgery, Heidelberg University Hospital, Heidelberg University, 69118 Heidelberg, Germany; Therese.Bormann@med.uni-heidelberg.de (T.B.); Steffen.Braun@med.uni-heidelberg.de (S.B.); Sebastian.Jaeger@med.uni-heidelberg.de (S.J.)

**Keywords:** total hip arthroplasty, implant deformation, acetabulum

## Abstract

Acetabular cup deformation may affect liner/cup congruency, clearance and/or osseointegration. It is unclear, whether deformation of the acetabular components occurs during load and to what extent. To evaluate this, revision multi-hole cups were implanted into six cadaver hemipelvises in two scenarios: without acetabular defect (ND); with a large acetabular defect (LD) that was treated with an augment. In the LD scenario, the cup and augment were attached to the bone and each other with screws. Subsequently, the implanted hemipelvises were loaded under a physiologic partial-weight-bearing modality. The deformation of the acetabular components was determined using a best-fit algorithm. The statistical evaluation involved repeated-measures ANOVA. The mean elastic distension of the ND cup was 292.9 µm (SD 12.2 µm); in the LD scenario, 43.7 µm (SD 11.2 µm); the mean maximal augment distension was 79.6 µm (SD 21.6 µm). A significant difference between the maximal distension of the cups in both scenarios was noted (F(1, 10) = 11.404; *p* = 0.007). No significant difference was noted between the compression of the ND and LD cups, nor between LD cups and LD augments. The LD cup displayed significantly lower elastic distension than the ND cup, most likely due to increased stiffness from the affixed augment and screw fixation.

## 1. Introduction

Total hip arthroplasty (THA) is considered to be one of the most successful operations performed in orthopedic surgery and the treatment of choice for end-stage osteoarthritis of the hip [1]. Consequently, the frequency of hip joint arthroplasty continues to increase worldwide. In 2007 Kurtz et al. estimated that the demand for primary total hip arthroplasty in the USA will increase by 174% to 572,000 by 2030 [2]. Concurrently they projected that the demand for revision THA would increase between 2005 and 2030 from 40,800 to 96,700 procedures representing an increase of 137% [2]. Generally, the longevity of revision THA is less than that of primary THA [3]. Lie et al. in 2004 analyzed 4762 revisions reported to the Norwegian Arthroplasty Register with a mean follow up of 3.2 years and found a 26% risk of failure after 10 years for cases without prior infection [3].

Cementless acetabular components have achieved widespread acceptance in THA as a result of their improved and reliable long-term results [4]. Primary stability is achieved through press-fit fixation that requires 1–3 mm under-reaming of the acetabular cavity and forceful impaction [5]. The forces utilized are substantial and generally result in some degree of deformation of the metal cup [6,7], particularly when the cup is thin-walled and of large diameter [8]. Meding et al. have shown that implant deformation resulting from the implantation process is non-uniform and results in diametrical pinching close to the implant rim, which is ascribed to impact pressure against the ischial and ileal columns of the acetabulum [8]. Prior studies have focused on assessing the pattern and degree of deformation resulting from the implantation process [7,9,10,11,12,13,14]. A few studies have assessed deformation after press-fit implantation and load application [15,16], however, these studies did not investigate cup deformation during load on the cup, as would be the case once the patient moves his/her hip.

The goal of our study was to evaluate and compare the elastic deformation of in-vitro titanium press-fit cups in two revision THA scenarios during subjection to cyclic gait loading for varying time periods. One sample group consisted of cadaver bone implanted with only a revision Gription cup (Pinnacle Multihole with Gription coating, Depuy/Synthes) (GC), whereas the second group was implanted with a revision Gription cup (GCS) and Gription augment (GAS) construct (Depuy/Synthes) both fixed to the pelvis with screws. We considered the diametrical change of the component rim during cyclic loading to relate to and reflect elastic deformation of the component that could potentially influence stable seating of the implant and the bone/implant apposition. This could contribute to micromotion on the implant-bone interface and between the cup and modular liner, respectively, which in turn might affect the osseointegration of the implant or influence the backside wear of the liner.

## 2. Materials and Methods

This study was approved by the local ethics committee (Ethikkommission Medizinische Fakultät Heidelberg, S309/2011).

Six fresh frozen cadaver hemipelvises were thawed at room temperature, dissected free of soft tissue. BMD measurements were done on attached femoral neck fragments prior to their removal from the hemipelvises. BMD was evaluated using Dual X-ray Absorptiometry (DXA) (QDR-2000 DXA densitometer; Hologic Inc, Waltham, MA, USA) and AP radiographs were also obtained on all specimens for pre-operative planning, and to exclude relevant pathology.

The average donor age was 78 years (range 51 to 96 years); the average donor mass was 66 kg (range 49 to 86 kg); and the average donor height was 171 cm (range 147.3 to 177.8 cm) resulting in a mean BMI of 23 kg/m^2^ (range 19.0 to 28.9 kg/m^2^). The mean bone mineral density, BMD, was 0.8 g/cm^2^ (range 0.641 to 0.924 g/cm^2^). Cadaver pelvises with a BMD below 0.6 g/cm^2^ were excluded.

We compared two component scenarios that are frequently used in revision surgery.

### 2.1. Scenario 1: Revision Cup without Substantial Bony Defect (ND)

In the first scenario, a revision Gription cup (GC) (Pinnacle^®^ Multihole with Gription^®^ coating, DePuy Synthes Companies, Warsaw, IN, USA) was implanted press-fit in six hemipelvises without bone defects according to manufacturer recommendations as described in previous studies [17,18]. The acetabula were reamed in a concentric fashion in 2 mm increments removing all cartilage. The last reamer used was 1 mm smaller than the corresponding cup size, allowing a press-fit insertion of the cup. The cups were implanted at 45° of inclination and 15° of anteversion. Cup sizes of 50, 52, 54, 56, and 58 mm (58 mm cup was implanted in two cases) were used to correspond to the respective acetabular cavity. No additional screws were used as the press-fit allows instant stability of the cup in the bone.

### 2.2. Scenario 2: Revision Cup with Large Bony Defect Treated Using an Augment (LD)

In the second scenario, the same six hemipelvises received a Paprosky 2b defect of 10 mm depth, which was created in a standardized manner at the posterolateral aspect of the acetabulum. 30% of the circumference of the rim was involved and the edge of the defect bordered on the anterior inferior iliac spine. The defects were subsequently covered with a 10 mm Gription augment (GAS), which was fixed to the host bone with two 5.5 mm × 30 mm screws, the so-called “augment-first technique”. A Gription cup (GCS, Gription Cup with Screws) was then fixed to the host bone using three 6.5 mm × 30 mm screws and to the augment with one 6.5 mm × 15 mm screw. Cup diameters referred to 50, 52, 54, 56, 58 and 58 mm. No cement was used. Marathon^®^ cross-linked polyethylene liners (DePuy Synthes Companies, Warsaw, IN, USA) corresponding to the respective cup and a 28 mm diameter metal head were used in all cases. THA’s were performed by a highly trained and experienced surgeon (RGB). Post-operative radiographs were obtained and confirmed the positioning of the implants and exclude fractures in all cases.

The implanted hemi-pelvises were fixated in a container using polyurethane foam (RenCast FC 53 A/B, Goessel + Pfaff GmbH, Karlskron/Brautlach, Germany) and integrated into a custom-made multi-axial testing machine (TD, testing device). This customized multi-axial testing machine enabled us to apply the changing loads and force vectors generated during a normal gait cycle as described in prior studies [17,18].

Loads applied were taken from the Bergmann et al. OrthoLoad data set [19]. Bergmann et al. [20] divided the gait cycle into phases and for each phase load components in the x, y and z axes were given according to a defined coordinate system. Using the load data (*F_x_, F_y_, F_z_*) and their respective angles, the orientation of the resultant force vectors during normal gait could be replicated. Using this data in our testing machine, the magnitude and direction of the force vectors were controlled by the MTS regulator (MTS headquarters, 14000 Technology Drive, Eden Prairie, MN, USA).

Utilizing our TD, our specimens were subjected to our adjusted loads in a cyclic physiologic manner that mimicked the normal gait cycle with the difference that we limited the applied load to 30% of that experienced in the normal gait cycle. We chose 30% of the normal load as an estimate of the partial weight-bearing allowed in patients during the immediate postoperative phase of revision hip arthroplasty. The loads we applied varied from a maximum of 69.93% to a minimum of 8.71% of body weight compared to the respective values at 233.1% and 29.02%, which have been determined with full weight bearing in the normal gait cycle [20,21]. One thousand load cycles were carried out at 1 Hz.

**Figure 1:**
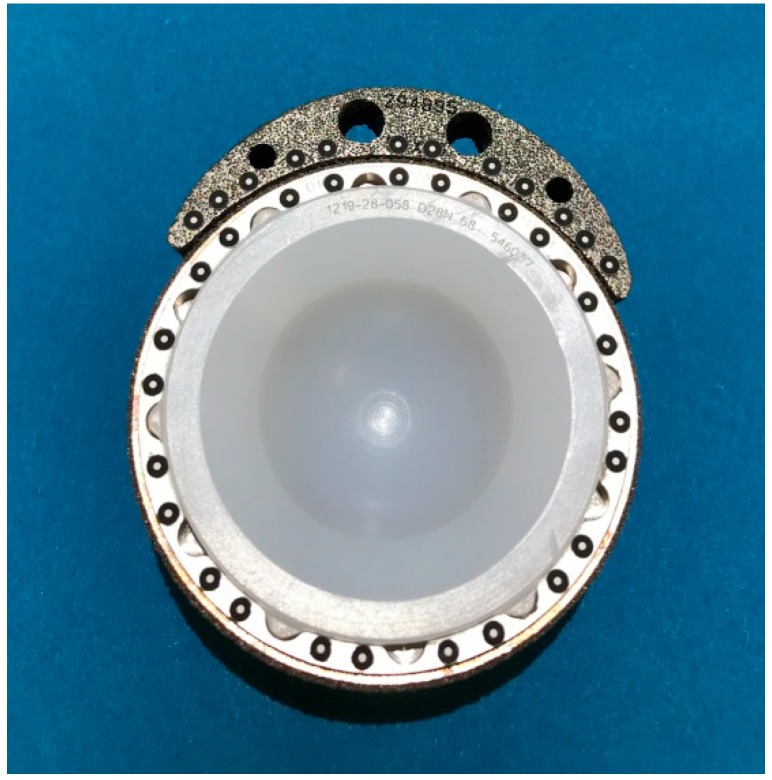
Photograph of acetabular cup, augment and liner. Note the attached optical marker points along the rim of the cup and augment.

Marker points (size 0.8 mm, GOM Gmbh, Braunschweig, Germany) trackable by an optical measuring system were placed around the circumference of the cup rim. They were also placed along the rim of the augment that constituted 30% of the entire cup circumference (see Figure 1).

**Figure 2:**
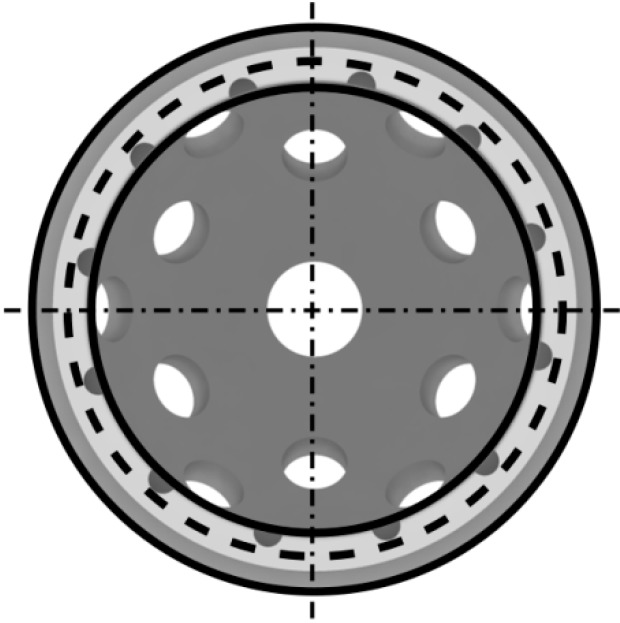
Representation of best-fit circle, denoted by the dotted lines around the rim of the acetabular cup, which was calculated by the relative motion of the optical markers.

The measurements were taken at 0, 50, 100, 200, 400, 600, 800, and 1000 cycles using a frame rate of 15Hz. For each set of load cycles, we used the optical readings from the cup rim and augment marker points, respectively, to calculate best-fit circles by utilizing the Best Fit Algorithm that then enabled us to measure the maximal and minimal circle diameters at each set of load cycles (see Figure 2) [22,23]. We considered elastic deformation to be the change in diameter of these “best-fit” circles from the circle diameter measured initially after implantation without load, which was calculated from the positional changes of the rim markers. Mean maximum and minimum diameter changes (mean maximum and minimum deformation) were calculated for both GC (measurement 1), and for GCS and GAS (measurement 2) construct groups for each of 0–1000 cycle sets. The results for the three groups were then compared.

## 3. Statistics

The data were evaluated descriptively using the arithmetic mean, standard deviation, minimum and maximum. A repeated-measures analysis of variance (ANOVA) was performed to test for significant differences for the parameter of deformation (primary cup vs. cup with screws and cup with screws vs. augment). Prior to data analysis, the normal distribution of the data was evaluated using a Shapiro–Wilk test, which was chosen over other statistical tests of normal distribution since a prior study has shown that this test provides more power given a known significance than other tests of normal distribution [24]. Subsequently, the homogeneity of variance was verified using the Levene test, which is a prerequisite for ANOVA. The results allowed for the use of the ANOVA test. The Greenhouse–Geisser adjustment was used to correct for violations of sphericity. The data were analyzed using SPSS 25 (IBM, Armonk, New York, NY, USA).

## 4. Results

**Figure 3:**
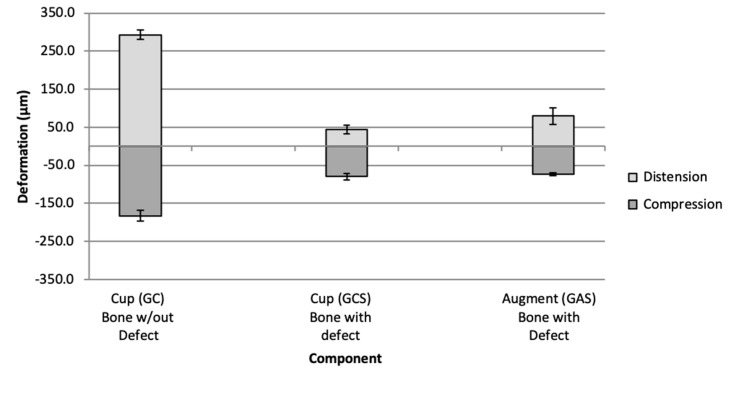
Bar graph of mean elastic deformation (distension and distension) of GC (no bony defect; scenario 1) and GCS and GAS (with bony defect; scenario 2).

The results are displayed below in Table 1 and Figure 3.

Comparison of the compression and distension deformation between primary and revision cup:

The cyclical loading showed no statistically significant effect under the standardized loading conditions with regards to the compression deformation, F(3.027, 30.266) = 0.484, *p* = 0.698. There was no statistically significant difference for the compression cup deformation between the primary and revision groups, F(1, 10) = 1.740, *p* = 0.217, yet a tendency for higher elastic deformation of the ND cup (GC) can be found.

The duration of load (i.e., the later load cycles) had no significant influence on the distension deformation, F(1.718, 17.176) = 0.368, *p* = 0.666. The repeated measures ANOVA with Greenhouse-Geisser correction determined that the difference in distension deformation of the cups in scenario 1 (No Defect) and scenario 2 (Large Defect) was statistically significant, F(1, 10) = 11.404, *p* = 0.007.

Comparison of the compression and distension deformation between the revision cup and augment:

Under the standardized loading conditions the cyclical loading showed no statistically significant effect with regards to the compression deformation, F(1.889, 18.892) = 1.048, *p* = 0.367. There was no statistically significant difference for the compression deformation between the revision cup and augment, F(1, 10) = 0.015, *p* = 0.904.

The duration of load (the later load cycles) had no significant influence on the distension deformation, F(1.515, 15.152) = 1.104, *p* = 0.340. The repeated measures ANOVA with Greenhouse-Geisser correction determined that there was no statistically significant difference between LD cup (GCS) and augment (GAS) with regards to the distension deformation of each, F(1, 10) = 0.389, *p* = 0.547.

The results are displayed in Table 1 and Figure 3.

## 5. Discussion

A large body of existing research has played an important role in the current success of both primary and revision THA, and resulted in a steadily increasing number of procedures and a decreasing age of patients [25,26]. Loosening and dislocation have been identified as the major causes of implant failure [26,27,28] and have been the focus of most research. Micro-motion at the bone/implant interface with subsequent particle production, tissue reaction, and osteolysis has been well documented [17,29,30,31,32]. Primary stability of the implant is recognized as critically important for ultimate surgical success [33,34,35]. Early osseointegration of all porous metal implants requires only minimal relative motion between implant and host bone. Micro-motion or bone/implant gap size of up to 50 µm has been shown to result in successful osseointegration, but above 150 µm there is attachment by fibrous tissue [33,36,37,38].

In contrast to studies of micromotion and other contributing factors leading to implant failure, implant deformation has received relatively little research attention. Existing studies have focused almost exclusively on deformation changes that occur during the process of implantation, and have disregarded deformation occurring afterward. The deformation of press-fit acetabular cups into 1–3 mm under-reamed sockets has been documented in several prior studies [8,37]. Prior researchers noted that the acetabular bone is most dense at the anterosuperior and posteroinferior margins (ileal and ischial columns) and the pubic area, constituting 3-point support [28]. The ileal and ischial columns are the most unyielding foci of the acetabular rim during the implantation process [12,28], and post-implantation provide the most bone/implant contact, foci of greatest load transfer and ultimately the most support for the implant [6,28]. Studies have shown that contact at the pelvic rim/implant area constitutes 25–50% of the total host bone/ implant apposition [9,36]. The host bone implant apposition decreases from rim to pole [39]. It has been found that even under ideal circumstances, total bone/implant apposition is never achieved and is unnecessary for successful osseointegration [9]. Cadaver studies of previously well-functioning components have shown that bone/implant contact areas have varied widely in extent [36]. The pelvic rim is also the area of greatest implant support during weight-bearing/gait loading [28].

During forceful press-fit implantation (previously measured at 400 N with porous titanium acetabular cups in-vivo) [8,37], the ileal and ischial columns exert a pinching effect on the cup component [7,9,12,14,35,37]. This causes the cup to assume a hemi-elliptical rather than a hemispherical shape that can lead to incongruity, diminished apposition and increased gap areas at the bone/implant interface [9,11,35]. These gaps, if excessive, can be associated with a number of negative consequences, including improper depth and angle of implant seating with subsequent potential dislocation, increased micro-motion at the bone/implant interface [35], facilitation of particle accumulation and increased tissue fluid, impaired liner insertion secondary to distortion of the cup locking mechanism and diminished clearance that adversely affects joint lubrication and liner wear [8,9,11,35,37,40]. Other factors that have been shown to influence the degree of cup deformation are the reaming process prior to implantation and the geometry of the acetabular cavity, characteristics of the implanted cup, bone density/hardness, the force applied during implantation and the seating of the cup [8,9,37,41,42]. Prior studies have shown that manual reaming results in a cavity that is usually slightly larger than the last reaming instrument and is hemi-elliptical in shape as a result of the varying degrees of stiffness throughout the host acetabular cavity [7,9,14,42]. Lin et al. found large errors in hand-reamed cavities [34]. Therefore, careful reaming of the acetabular cavity and accurate cup seating have been identified as significant modifiable factors in reducing the degree of implant deformation [9] that occurs during the implantation process, and we hypothesize that they may also influence the degree of elastic deformation that is the focus of our study. Characteristics of the implant that have been found to influence the potential to deform are the type of metal used, and diameter and thickness of the cup. Meding et al. found that titanium cups deform more than those of CoCr, and increasing the diameter and thinness of the wall are associated with increased potential to deform [8,35].

The focus and methodology of our current study differ in several respects from all prior studies of deformation. Our study does not evaluate deformation occurring from the process of implantation, but looks at elastic deformation occurring as a result of cyclic loading applied several days post-implantation. We utilized cadaver bone rather than the synthetic bone to permit the most realistic testing scenario, and measurements were made several days after implantation to minimize the elliptical distortion of the implantation process since it has been demonstrated that for several days post-implantation there is a visco-elastic relaxation of the pelvic bone and implant that reduces the deformation of titanium components [7]. We focused on the rim since it has been shown to be the site of the most bone/implant apposition and the region of maximal loading with gait [6,28]. Since our methodology utilized the derivation of a “best-fit circle” based on an assumption of symmetry of the acetabular implant, our results are not direct measurements of implant deformation or of gap size and can only be used for comparison of our two study groups.

Our results showed that all our study specimens displayed some elastic deformation during cyclic loading, but there was a distinct difference between the two groups. We found statistically significantly more elastic deformation in Scenario 1 (revision cup only, lack of bony defect) than in Scenario 2 (revision cup plus augment and screws implanted into a Paprosky 2b defect). Values for the revision cup with screws and values for the augment with screws were not significantly different from each other, indicating that cup plus augment and screws tend to function as a unit.

For all specimens, maximal elastic deformation (compression and distension) was reached after approximately 50 cycles of loading with no significant additional deformation noted with increased cycles.

We hypothesize that the addition of screws and screws plus augment effectively increases the rigidity of the construct, which is consistent with findings of prior studies on deformation that occurred secondary to implantation, which showed that rigid CoCr cups deformed less than titanium cups [8]. Additionally, the compression of the cup and augment in scenario 2 proved more substantial than the distension.

Our results are clinically significant in several ways. The use of ancillary screws decreased the degree of elastic deformation of the implant rim, allowing better bone/implant apposition, reduced gap areas, and potentially improved osseointegration. The presence of an augment did not negatively impact the degree of elastic deformation, and the cup/augment construct effectively functioned as a unit. Our findings also indicate that cyclic loading that mimics normal gait is associated with increased elastic deformation and validates prior findings [9,35]. We propose that the elastic deformation we identified, and the deformation of implantation may both be potential influencing factors in the occurrence of backside wear of modular liners. Furthermore, acetabular component deformation during load may affect clearance at the articulating interface and may play a role in the rare, albeit relevant liner dislocation. One case series reported on 23 liner dissociations after Pinnacle implantation [43], which may be a result of overly elastic cups.

Cup diameter and wall thickness have repeatedly been shown to affect cup deformation. Large (jumbo) porous cups are an alternative option to cup/augment combination in revision THA associated with deficient bone stock and acetabular defects, and their large diameter and thin walls predispose to increased deformation, although ancillary screw fixation may limit this deformation. Oversized porous cups have been reported to have an increased risk of dislocation of multifactorial cause [6,8,44]. Deformation of the cups has not been considered a contributing factor but may be worthy of further consideration.

## 6. Limitations

Our study has several limitations. The cohorts were small, and only two revision constructs were assessed, the first without substantial osseous defect, and the second using a substantial (Paprosky 2b) bony defect.

The optical markers covered only half the implant rim, since the remaining rim was concealed from the optical cameras by the moving prosthesis neck—we assumed that the cup rim was symmetrical. Our methodology utilized the optical marker data to derive a “best-fit circle” and the change of circle diameter was considered to reflect elastic deformation. Therefore, our results are not a direct measure of deformation or bone/implant gap regions, but serve only as an indirect indicator, and can only be used as a means of comparison between our two study groups. This is the first study to utilize this methodology and evaluate deformation during loading, and there are therefore no currently existing comparable data for verification.

We utilized cadaver bone since the polyurethane models were validated only for deformation occurring during implantation [8] and we considered cadaver bone to be more physiologic. However, it lacks some of the viscoelastic properties of live bone, and may not reflect the clinical scenario [12,36].

There is tentative evidence that hardness of bone, related to BMD, necessitates increased compressive force for implantation that may affect results. Our cadaver sample was from older patients with a mean BMD of 0.8g/cm^2^ (range 0.641 to 0.924 g/cm^2^) and values beyond this range may have different results.

In our experimental set-up, only Paprosky 2b defects were examined, and defects of other grades may have different results.

Multi-hole cups have been shown to present more deformation than single-hole cups during implantation [16], which allows one to assume that a similar effect may be seen under dynamic loading after implantation. For the purpose of comparability, a multi-hole cup was used in both scenarios, although this type of cup would tend not to be used clinically in a scenario without a bony defect.

Variations in surgical technique cannot be excluded although the same highly experienced surgeon (RGB) performed all implantations with supplied manufacturers tools.

In conclusion, our in-vitro study utilizing revision constructs in cadaver bone is the first to compare the elastic deformation occurring at the rim of two different implant constructs during cyclic loading that replicates the limited loading of normal gait as experienced in the early postoperative period. Our results show that the use of adjunctive screws significantly decreases the degree of elastic deformation under these conditions, and the inclusion of an augment does not adversely impact the degree of elastic deformation.

## Figures and Tables

**Table 1:** Mean elastic deformation (µm) for scenario 1 (GC) and scenario 2 (GCS and GAS) during all cycle sets.

Deformation (µm)	Scenario 1 without Defect	Scenario 2 with Paprosky 2b Defect	
	Cup (GC)	Cup (GCS)	Augment (GAS)
Mean Compression ± SD	−182.4 ± 15.0	−79.9 ± 9.2	−73.2 ± 4.3
Mean Distension ± SD	292.9 ± 12.2	43.7 ± 11.2	79.6 ± 21.6
Overall Deformation	475.3	123.6	152.8

## References

[1] Learmonth I.D., Young C., Rorabeck C. (2007). The operation of the century: Total hip replacement. Lancet.

[2] Kurtz S., Ong K., Lau E., Mowat F., Halpern M. (2007). Projections of primary and revision hip and knee arthroplasty in the United States from 2005 to 2030. J. Bone Joint Surg. Am..

[3] Lie S.A., Havelin L.I., Furnes O.N., Engesaeter L.B., Vollset S.E. (2004). Failure rates for 4762 revision total hip arthroplasties in the Norwegian Arthroplasty Register. J. Bone Joint Surg. Br..

[4] Ng F.Y., Zhu Y., Chiu K.Y. (2007). Cementless acetabular component inserted without screws—The effect of immediate weight-bearing. Int. Orthop..

[5] Kroeber M., Ries M.D., Suzuki Y., Renowitzky G., Ashford F., Lotz J. (2002). Impact biomechanics and pelvic deformation during insertion of press-fit acetabular cups. J. Arthroplast..

[6] Bone M.C., Dold P., Flohr M., Preuss R., Joyce T.J., Deehan D., Holland J. (2013). A novel method for measuring acetabular cup deformation in cadavers. Proc. Inst. Mech. Eng. Part H.

[7] Springer B.D., Habet N.A., Griffin W.L., Nanson C.J., Davies M.A. (2012). Deformation of 1-piece metal acetabular components. J. Arthroplast..

[8] Meding J.B., Small S.R., Jones M.E., Berend M.E., Ritter M.A. (2013). Acetabular cup design influences deformational response in total hip arthroplasty. Clin. Orthop. Relat. Res..

[9] Ong K.L., Lehman J., Notz W.I., Santner T.J., Bartel D.L. (2006). Acetabular cup geometry and bone-implant interference have more influence on initial periprosthetic joint space than joint loading and surgical cup insertion. J. Biomech. Eng..

[10] Bone M.C., Dold P., Flohr M., Preuss R., Joyce T.J., Aspden R.M., Holland J., Deehan D. (2015). The influence of the strength of bone on the deformation of acetabular shells: A laboratory experiment in cadavers. Bone Joint J..

[11] Panjeton G.D., Kim S.E., Chang K., Palm L.S., Ifju P.G. (2017). Deformation of the Zurich cementless acetabular cup caused by implantation in a canine cadaver model. Vet. Surg..

[12] Markel D., Day J., Siskey R., Liepins I., Kurtz S., Ong K. (2011). Deformation of metal-backed acetabular components and the impact of liner thickness in a cadaveric model. Int. Orthop..

[13] Liu F., Chen Z., Gu Y., Wang Q., Cui W., Fan W. (2012). Deformation of the Durom acetabular component and its impact on tribology in a cadaveric model—A simulator study. PLoS ONE.

[14] Fritsche A., Bialek K., Mittelmeier W., Simnacher M., Fethke K., Wree A., Bader R. (2008). Experimental investigations of the insertion and deformation behavior of press-fit and threaded acetabular cups for total hip replacement. J. Orthop. Sci..

[15] Ong K.L., Rundell S., Liepins I., Laurent R., Markel D., Kurtz S.M. (2009). Biomechanical modeling of acetabular component polyethylene stresses, fracture risk, and wear rate following press-fit implantation. J. Orthop. Res..

[16] Messer-Hannemann P., Campbell G.M., Morlock M.M. (2019). Deformation of acetabular press-fit cups: Influence of design and surgical factors. Clin. Biomech. (Bristol, Avon).

[17] Beckmann N.A., Bitsch R.G., Janoszka M.B., Klotz M.C., Bruckner T., Jaeger S. (2018). Treatment of High-Grade Acetabular Defects: Do Porous Titanium Cups Provide Better Stability Than Traditional Titanium Cups When Combined With an Augment?. J. Arthroplast..

[18] Beckmann N.A., Jaeger S., Janoszka M.B., Klotz M.C., Bruckner T., Bitsch R.G. (2018). Comparison of the Primary Stability of a Porous Coated Acetabular Revision Cup With a Standard Cup. J. Arthroplast..

[19] OrthoLoad. http://www.OrthoLoad.com.

[20] Bergmann G., Graichen F., Rohlmann A. (1993). Hip joint loading during walking and running, measured in two patients. J. Biomech..

[21] Bergmann G., Graichen F., Rohlmann A., Bender A., Heinlein B., Duda G.N., Heller M.O., Morlock M.M. (2010). Realistic loads for testing hip implants. Biomed. Mater. Eng..

[22] Weißmann V., Boss C., Schulze C., Hansmann H., Bader R. (2018). Experimental Characterization of the Primary Stability of Acetabular Press-Fit Cups with Open-Porous Load-Bearing Structures on the Surface Layer. Metals.

[23] Mueller U., Lee C., Heisel C., Thomsen M., Bitsch R.G., Kretzer J.P. (2016). Failure of Polyethylene Inlays in Cementless Total Hip Arthroplasty: A Retrieval Analysis. BioMed. Res. Int..

[24] Razali N.M., Yap B. (2011). Power Comparisons of Shapiro-Wilk, Kolmogorov-Smirnov, Lilliefors and Anderson-Darling Tests. J. Stat. Model. Anal..

[25] Ulrich S.D., Seyler T.M., Bennett D., Delanois R.E., Saleh K.J., Thongtrangan I., Kuskowski M., Cheng E.Y., Sharkey P.F., Parvizi J. (2008). Total hip arthroplasties: What are the reasons for revision?. Int. Orthop..

[26] Bozic K.J., Kurtz S.M., Lau E., Ong K., Vail T.P., Berry D.J. (2009). The epidemiology of revision total hip arthroplasty in the United States. J. Bone Joint Surg. Am..

[27] Widmer K.H., Zurfluh B., Morscher E.W. (2002). Load transfer and fixation mode of press-fit acetabular sockets. J. Arthroplast..

[28] Schmalzried T.P., Jasty M., Harris W.H. (1992). Periprosthetic bone loss in total hip arthroplasty. Polyethylene wear debris and the concept of the effective joint space. J. Bone Joint Surg. Am..

[29] Beckmann N.A., Bitsch R.G., Gondan M., Schonhoff M., Jaeger S. (2018). Comparison of the stability of three fixation techniques between porous metal acetabular components and augments. Bone Joint Res..

[30] Wirtz D.C., Niethard F.U. (1997). Etiology, diagnosis and therapy of aseptic hip prosthesis loosening—A status assessment. Z. Orthop. Ihre Grenzgeb..

[31] Aspenberg P., Herbertsson P. (1996). Periprosthetic bone resorption. Particles versus movement. J. Bone Joint Surg. Br..

[32] Pilliar R.M. (1987). Porous-surfaced metallic implants for orthopedic applications. J. Biomed. Mater. Res..

[33] Wetzel R., Simnacher M., Scheller G. (2005). Initial stability of press-fit acetabular cups—An in-vitro study. Biomed. Technol. (Berl).

[34] Lin Z.M., Meakins S., Morlock M.M., Parsons P., Hardaker C., Flett M., Isaac G. (2006). Deformation of press-fitted metallic resurfacing cups. Part 1: Experimental simulation. Proc. Inst. Mech. Eng. Part H.

[35] Squire M., Griffin W.L., Mason J.B., Peindl R.D., Odum S. (2006). Acetabular component deformation with press-fit fixation. J. Arthroplast..

[36] Schwartz J.T., Engh C.A., Forte M.R., Kukita Y., Grandia S.K. (1993). Evaluation of initial surface apposition in porous-coated acetabular components. Clin. Orthop. Relat. Res..

[37] Engh C.A., O’Connor D., Jasty M., McGovern T.F., Bobyn J.D., Harris W.H. (1992). Quantification of implant micromotion, strain shielding, and bone resorption with porous-coated anatomic medullary locking femoral prostheses. Clin. Orthop. Relat. Res..

[38] Pilliar R.M., Lee J.M., Maniatopoulos C. (1986). Observations on the effect of movement on bone ingrowth into porous-surfaced implants. Clin. Orthop. Relat. Res..

[39] MacKenzie J.R., Callaghan J.J., Pedersen D.R., Brown T.D. (1994). Areas of contact and extent of gaps with implantation of oversized acetabular components in total hip arthroplasty. Clin. Orthop. Relat. Res..

[40] Lachiewicz P.F., Watters T.S. (2016). The jumbo acetabular component for acetabular revision: Curtain Calls and Caveats. Bone Joint J..

[41] Spears I.R., Morlock M.M., Pfleiderer M., Schneider E., Hille E. (1999). The influence of friction and interference on the seating of a hemispherical press-fit cup: A finite element investigation. J. Biomech..

[42] Yew A., Jin Z.M., Donn A., Morlock M.M., Isaac G. (2006). Deformation of press-fitted metallic resurfacing cups. Part 2: Finite element simulation. Proc. Inst. Mech. Eng. Part H.

[43] Yun A., Koli E.N., Moreland J., Iorio R., Tilzey J.F., Mesko J.W., Lee G.C., Froimson M. (2016). Polyethylene Liner Dissociation Is a Complication of the DePuy Pinnacle Cup: A Report of 23 Cases. Clin. Orthop. Relat. Res..

[44] Dold P., Bone M.C., Flohr M., Preuss R., Joyce T.J., Deehan D., Holland J. (2014). Validation of an optical system to measure acetabular shell deformation in cadavers. Proc. Inst. Mech. Eng. Part H.

